# Associations between the injustice experience questionnaire and treatment term in patients with acute Whiplash-associated disorder in Japan: Comparison with Canadian data

**DOI:** 10.1371/journal.pone.0231077

**Published:** 2020-04-13

**Authors:** Kazuhiro Hayashi, Kenji Miki, Tatsunori Ikemoto, Takahiro Ushida, Masahiko Shibata

**Affiliations:** 1 Pain Medicine and Research Information Center, Nonprofit Organization, Nagakute, Japan; 2 Multidisciplinary Pain Center, Aichi Medical University, Nagakute, Japan; 3 Department of Rehabilitation, Aichi Medical University Hospital, Nagakute, Japan; 4 Center for Pain Management, Hayaishi Hospital, Osaka, Japan; 5 Faculty of Health Science, Osaka Yukioka College of Health Science, Osaka, Japan; 6 Department of Orthopedic Surgery, Aichi Medical University, Nagakute, Japan; 7 Institute of Physical Fitness, Sports Medicine and Rehabilitation, Aichi Medical University, Nagakute, Japan; 8 Department of Health Science, Naragakuen University, Nara, Japan; Tokyo Metropolitan Institute of Medical Science, JAPAN

## Abstract

**Introduction:**

This study aimed to investigate the differences in the Injustice Experience Questionnaire (IEQ) scores during the early period after the diagnosis of Whiplash-associated disorder (WAD) between Japanese and Canadian samples, and the associations between the IEQ scores and treatment terms in Japanese patients with acute WAD.

**Methods:**

We used secondary data for the IEQ scores of Canadian patients with acute WAD. In Japan, we collected data from 85 consecutively enrolled patients with acute WAD, and their treatment terms were collected; these referred to the number of days between the date of injury and the closure date of the insurance claim and the number of treatment visits. Before treatment, the Numeric Rating Scale, Neck Disability Index, Hospital Anxiety and Depression Scale, IEQ, and Euro Quality of Life five-dimensional questionnaire were administered. The variables were subjected to multivariate analysis with each treatment term.

**Results:**

The IEQ scores were higher in Japan than in Canada. Through multiple regression analysis, IEQ scores were independently correlated with treatment terms. The optimal cutoff point of the IEQ scores for a prolonged treatment term was 21 and 22 points, respectively.

**Conclusions:**

The IEQ scores were associated with treatment terms in patients with acute WAD in Japan.

## Introduction

The Whiplash-associated disorder (WAD) encompasses the bony or soft-tissue injuries resulted from acceleration or deceleration forces applied to the neck, such as those associated with motor vehicle accidents. [1] The Quebec Task Force classifies patients with WAD based on the severity of signs and symptoms, [1] as follows: Grade 0, no complaints about the neck and no physical signs; Grade 1, pain complaint around the neck, stiffness or tenderness in the region, and no physical signs; Grade 2, pain complaint, musculoskeletal signs—including decreased range of motion and point tenderness; Grade 3, pain complaint, musculoskeletal signs, neurological signs—including decreased/absent deep tendon reflexes, muscle weakness, and sensory deficits; Grade 4, pain complaint and neck fracture or dislocation. Further, more than 80% of patients with WAD are classified as less than Grade 2. [1] Patients with a WAD classification less than Grade 2 are commonly managed by non-invasive treatments, [1,2] although the level of evidence related to the condition is reportedly low/very low. [1]

Usually, pain and disability symptoms related to the WAD reduce rapidly in the initial month after the injury, but show little improvement 3 months after. [3] Treatment terms are a type of document often used as an official declaration of the number of days that passed until insurance claim closure and the number of treatment visits the patient has undergone. [4–6] The number of days that passed until insurance claim closure refers to the days between the date of injury and the insurance claim closure date. [4,5] This claim closure is validated as a marker of health recovery given its relationship to clinically important levels of improvement in pain intensity, physical function, and psychological symptoms. [4] In Japan, the number of treatment visits is also one of the composing elements of the treatment terms document because the government uses these data to measure the corresponding compensation for treatment related to insurance claim closure. [5,6]

A systematic review of acute WAD indicates that the treatment term is associated with post-injury symptoms and some psychosocial factors, not injuries related to physical or mechanical factors. [7] The same review suggests that, although there are associations between the prognosis of WAD and psychological variables (including coping behavior, general psychological distress, and depressive mood), current evidence is inconsistent in this regard. [7]

Recently, perceived injustice related to health care has been proposed as a psychological construct. [8,9] It is defined as a multidimensional construct comprising elements that assess loss severity, loss irreparability, blame, and sense of unfairness. [8,9] The Injustice Experience Questionnaire (IEQ) construes perceived injustice as an appraisal cognition, and it comprises four subscales correspondent to the aforementioned elements: loss severity (“Most people don’t understand the severity of my condition”), blame (“I am suffering because of someone else’s negligence”), a sense of unfairness (“It all seems so unfair”), and loss irreparability (“My life will never be the same”). [8,9] The IEQ scores are high in patients who believe that the liability for their injury rests in another person, [10] experience pain for a long duration, [10] get injured in a motor vehicle accident, [9] have severe pain, [9] and psychological disturbance. [9] In patients with sub-acute or chronic WAD, the IEQ scores associate with pain severity, [11] displays of pain behavior, [12] work disability, [11,13] depressive symptoms, [14] and post-traumatic stress symptoms. [15]

To date, only one study in Canada has investigated the association between IEQ scores and treatment terms among patients with acute WAD (*i*.*e*. < 3 months with the condition) injured in motor vehicle accidents; [16] this study suggests IEQ scores do not serve as predicting factors of prognostic for recovery during the early period after injury, but becomes associated with non-recovery at 6 months after injury and thereafter. [16] They have shown that the IEQ scores become elevated several months after the WAD injury among patients who have yet to recover from the condition. [16] Meanwhile, in Japan, a study among patients who suffered a motor vehicle accident and endured pain symptoms for less than one year showed their IEQ scores were high. [10] Pain-related psychological disturbances are often influenced by patients’ race, ethnicity, and culture. [17] Corroborating, previous studies have shown that symptomatic expectations among patients with WAD differ among different nationalities. [18–21] Notwithstanding, the impact of elevated IEQ scores on treatment terms during the early period after injury related to the WAD has yet to be investigated.

Therefore, this study aimed to investigate (1) the difference on the IEQ scores among Japanese and Canadian patients with WAD during the early period after the injury, and (2) the associations between the IEQ scores during the same period and treatment terms in Japanese patients with WAD.

## Methods

### Participants

The number of subjects was determined by a sample size estimation using the G*Power software (version 3.0.10; Franz Faul, Kiel University, Kiel, Germany). For a standardized effect size of 0.3, [7] an α-level of 0.05, and a power (1-β) of 0.80, the minimum number of subjects was estimated to be 82. Patients were recruited consecutively from a primary orthopedic clinic between July 2017 and February 2019.

The inclusion criteria were: being 18 years old or older, having acute injury (*i*.*e*. < 14 days) following the accident, a diagnosis of WAD Grade 1 or 2, [1] a single walk-in to the primary clinic, and ability to understand Japanese. Patients were followed up regardless of their treatment terms. Exclusion criteria were: having a previous neck injury, ongoing treatment for pain/pain-related symptoms, or lost to follow up.

These patients were treated through standardized care for the condition, including behavioral activation-based therapy (“act-as-usual,” education, and self-care, including regular exercise) and pharmacotherapy whenever necessary. [2] The physician instructed patients that they could stop the medication if their symptoms improved or any adverse events occurred during treatment.

This study was approved by the Ethics Committee of Hayaishi Hospital, and written informed consent was collected from all subjects prior to their participation.

### Measures

#### Demographic and motor vehicle accident data

Demographic data (age, sex, and presence/absence of affected body parts other than neck) were collected. Motor vehicle accident was assessed using level of collision impact and presence of liability for an accident: Level of collision impact was assessed through an interview, and questions were asked to confirm whether it was large or not. A large level of collision impact referred to damage configured not only by a simple bump into another vehicle but also by vehicles being turned over; by vehicles that, after the impact, were lying on their sides; or by the patient falling off of a motor cycle. Presence of liability for an accident referred to the presence of police and sometimes a lawyer in the process of dealing with the impact.

#### Treatment terms

Treatment terms referred to the number of days that passed until insurance claim closure and the number of treatment visits the patient has undergone. [4–6] The number of days that passed until insurance claim closure referred to the number of days passed between the date of injury and the date corresponding to the closure of the insurance claim. The closure of an insurance claim referred to the result of a negotiation between health-care providers, claimants, and sometimes a lawyer.

#### Pain and psychological variables

Pain and psychological variables were measured before treatment through the Numeric Rating Scale (NRS), [22,23] the Neck Disability Index (NDI), [24,25] the Hospital Anxiety and Depression Scale (HADS), [26,27] the Injustice Experience Questionnaire (IEQ), [9,10] and the Euro Quality of Life five-dimensional Questionnaire (EQ-5D). [28] All questionnaires had translated versions in Japanese.

- NRS [22,23]
  The NRS assesses pain severity, where 0 = no pain and 10 = worst pain imaginable.
- NDI [24,25]
  The 10-item NDI assesses neck pain symptoms. The questions cover pain, personal care, lifting, reading, headaches, concentration, work, driving, sleeping, and recreation. It is responded through a six-point scale (0–5), with scores ranging from 0–50.
- HADS [26,27]
  The 14-item HADS assesses anxiety and depression, and it has two separate subscales: the 7-item HADS-A (anxiety) and the 7-item HADS-D (depression). Responses are through a four-point scale (0–3), with scores for each subscale ranging from 0–21.
- IEQ [9,10]
  The IEQ assesses injury-related perceived injustice. Responders rate the frequency with which they experienced each of 12 pain-related perceptions. Responses are through a five-point scale (0–4), with scores ranging from 0–48.
- EQ-5D [28]
  The EQ-5D assesses subjective health-related quality of life. It has 5 subscales: mobility, self-care, usual activities, pain/discomfort, and anxiety/depression. Its scores range from −0.111–1.000, with negative scores meaning worse health than death, 0 meaning death, and 1.000 meaning a state of full health.

#### IEQ scores in Canada

The IEQ scores of Canadian patients with acute WAD were collected through the use of secondary/previous data. [16] Briefly, the cited study consecutively recruited patients with acute WAD Grade 1 or 2, who had no loss of consciousness, were 18 years old or older, who presented themselves in the clinic within 14 days of their collision, and who had a single walk-in to the primary care center. Similar to our study, the IEQ was applied before treatment.

#### Statistical analysis

Continuous data were expressed as means and standard deviations (SD). Non-continuous data (score data) were expressed as medians and interquartile ranges (IQR). The correlations between treatment terms and each variable were analyzed using Spearman’s rank correlation coefficient test. Dummy variables were used for categorical data. All graphs plotted the mean ± the SD of the mean.

Multiple regression analysis was used to investigate variables yielding P values <0.05 in the single regression analysis. Multi-collinearity of the variables was also assessed (when the correlation coefficient <0.7).

The optimal cutoff point of continuous data were determined using receiver operating characteristic (ROC) curve analyses, [29] and this served to predict a prolonged number of days until insurance claim closure (more than 3 months). Then, patients were categorized into two groups: the higher than the cutoff point and the lower than the cutoff point groups. The groups were compared using Chi-square test and Mann-Whitney U test.

Data were analyzed using IBM SPSS Statistics 25.0 software (IBM, Armonk, NY, USA). A p value of less than 0.05 was considered as statistically significant.

## Results

Ninety-three consecutive Japanese patients with acute WAD were included in our sample. Nonetheless, eight patients were lost to follow up, so 85 patients had their data analyzed. Of these, 29 (34%) were women (Table 1); the mean age was 46 years (SD, 19); the mean number of days passed until insurance claim closure was 106 days (SD, 100); and the number of treatment visits was 19 (SD, 26). Twenty-eight (33%) patients had injured their heads, 41 (48%) patients had injured their torso, and 29 (34%) patients had injured their limbs.

**Table 1:** Participant's characteristics (n = 85).

Women, n (%)	29 (34%)
Age (years)	46 (19)
Injured body parts in addition to the neck	
Injured their heads, n (%)	28 (33%)
Injured their torso, n (%)	41 (48%)
Injured their limbs, n (%)	29 (34%)
Large level of collision impact, n (%)	49 (58%)
Presence of liability for an accident, n (%)	17 (20%)
NRS (points)	5 [3–6]
NDI (points)	17 [12–23]
HADS-A (points)	6 [3–9]
HADS-D (points)	5 [3–10]
IEQ (points)	16 [0–29]
EQ-5D (points)	0.67 [0.59–0.72]
Number of days that passed until insurance claim closure (days)	106 (100)
Number of treatment visits (days)	19 (26)

NRS, Numeric Rating Scale; NDI, Neck Disability Index; HADS-A, Hospital Anxiety and Depression Scale (anxiety); HADS-D, Hospital Anxiety and Depression Scale (depression); IEQ, Injustice Experience Questionnaire; EQ-5D, Euro Qol five-dimensional questionnaire. Continuous data were expressed as means and standard deviations (SD). Non-continuous data (score data) were expressed as medians and interquartile ranges (IQR). Data from categorical variables are shown as number (%).

**Fig 1:**
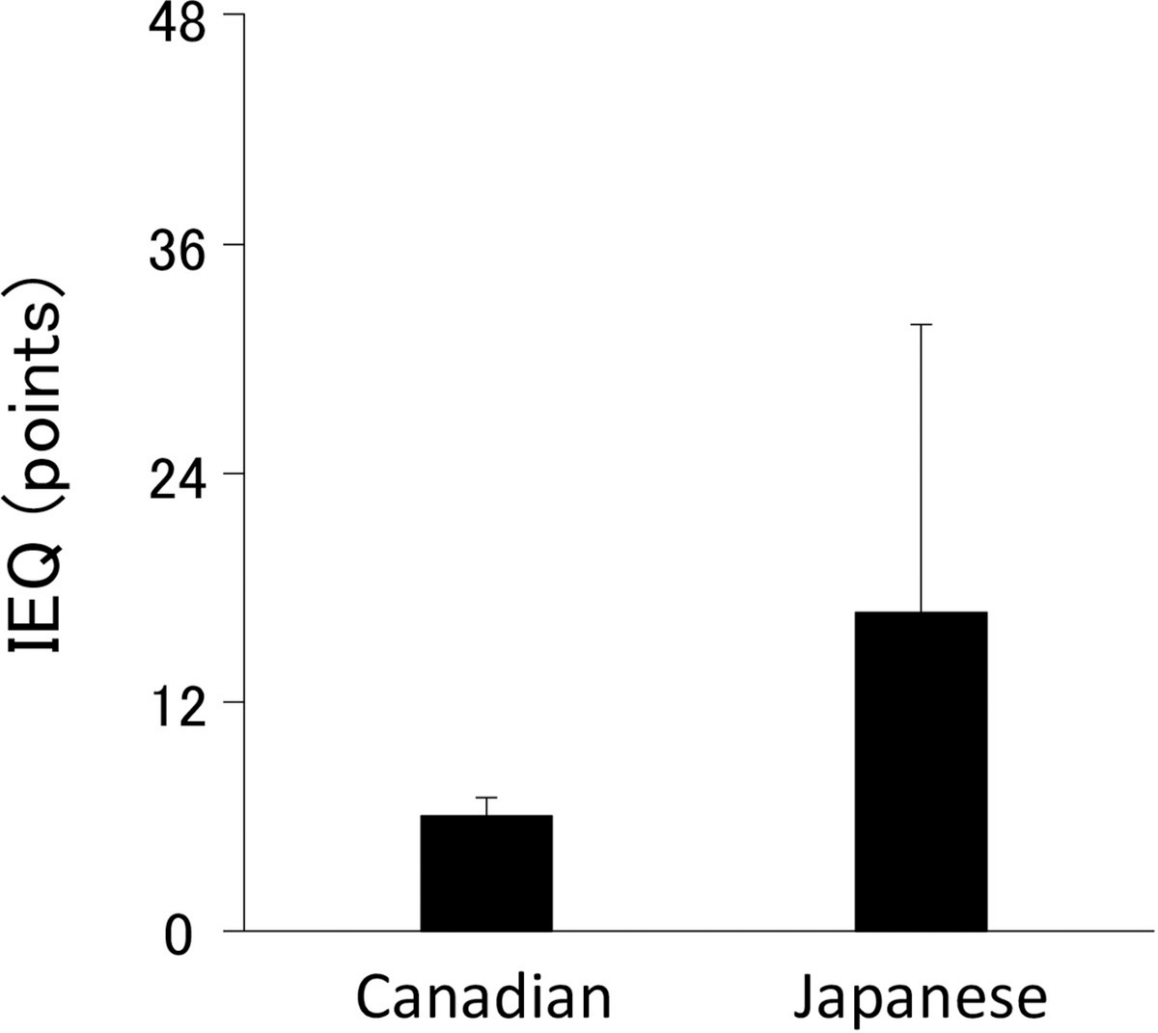
IEQ scores at before treatment. All graphs plotted the mean ± the SD of the mean. The IEQ mean scores of the Japanese sample were 2 points above the SD when compared to the mean scores of the Canadian sample. IEQ, Injustice Experience Questionnaire.

As shown in Fig 1, the IEQ mean scores of the Japanese sample (mean, 17; SD, 15) were 2 points above the SD when compared to the mean scores of the Canadian sample (mean, 6; SD, 1). [16 and communication with Robert Ferrari]

**Fig 2:**
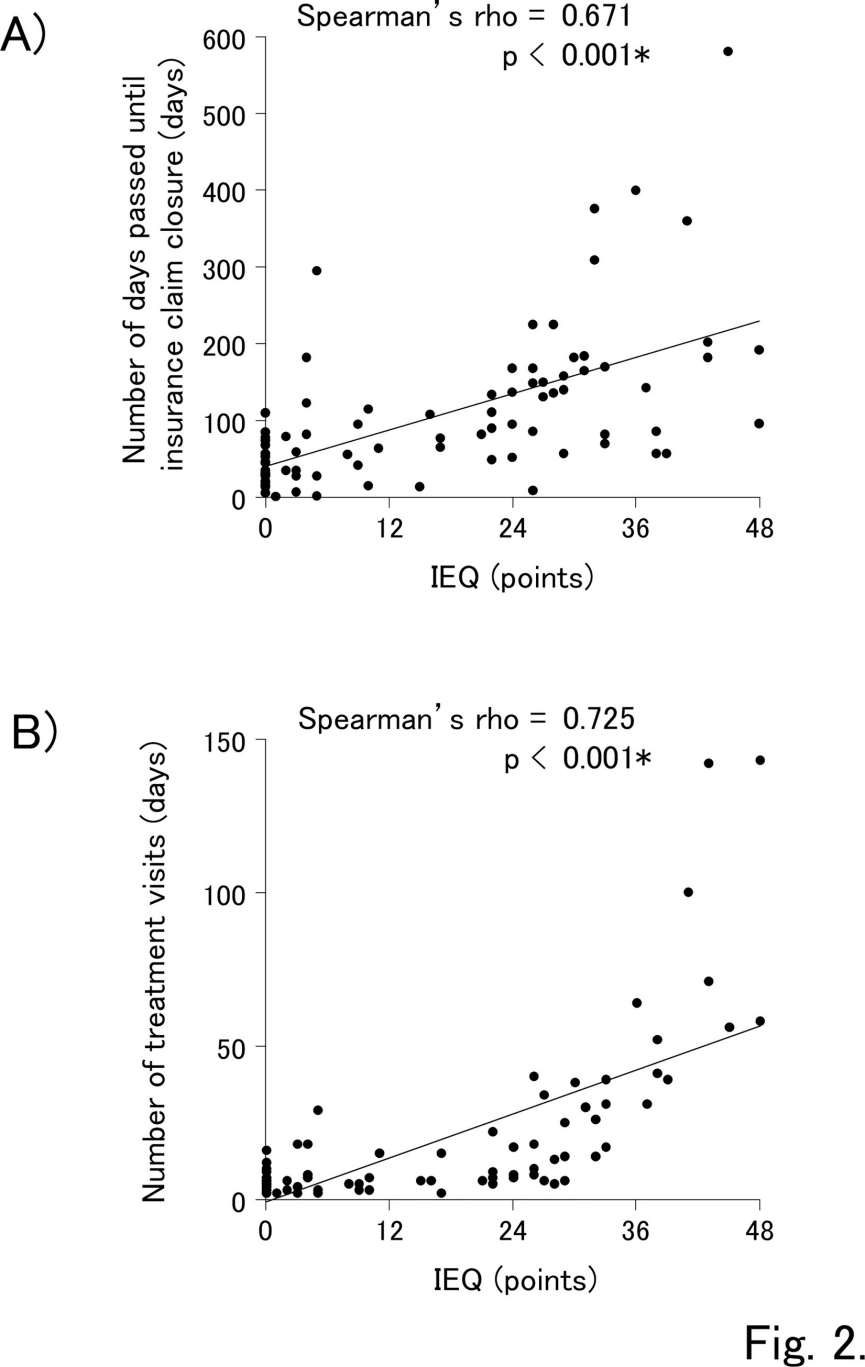
A). Correlation between the IEQ scores and the number of days that passed until insurance claim closure. B). Correlation between the IEQ scores and the number of treatment visits. The number of days that passed until insurance claim closure and the number of treatment visits were significantly associated with the IEQ score. IEQ, Injustice Experience Questionnaire. *Significance level was set at p < 0.05.

Correlations between each treatment term and the independent variables are shown in Table 2 and Fig 2. The number of days that passed until insurance claim closure and the number of treatment visits were significantly associated with the IEQ score (correlation coefficient > 0.6; p < 0.001). Patients with old age, and high HADS-A and HADS-D score groups tended to have higher number of days passed until insurance claim closure and higher number of treatment visits. Patients with large collision impact tended to have a shorter number of days passed until insurance claim closure, but it had no association with the number of treatment visits.

**Table 2:** Correlations between each treatment terms and the independent variables.

	Number of days that passed until insurance claim closure		Number of treatment visits	
	rs	p	rs	p
Women, n (%)	0.074	0.502	0.117	0.288
Age (years)	0.138	0.207	0.263	0.015*
Injured body parts in addition to the neck				
Injured their heads, n (%)	0.165	0.131	0.128	0.242
Injured their torso, n (%)	0.166	0.129	0.169	0.121
Injured their limbs, n (%)	0.170	0.119	0.074	0.501
Large level of collision impact, n (%)	-0.183	0.094	-0.087	0.431
Presence of liability for an accident, n (%)	-0.082	0.458	-0.026	0.814
NRS (points)	0.130	0.237	0.112	0.307
NDI (points)	0.177	0.104	0.159	0.147
HADS-A (points)	0.304	0.005*	0.191	0.080
HADS-D (points)	0.258	0.017*	0.262	0.015*
IEQ (points)	0.671	<0.001*	0.725	<0.001*
EQ-5D (points)	-0.167	0.127	-0.180	0.100

NRS, Numeric Rating Scale; NDI, Neck Disability Index; HADS-A, Hospital Anxiety and Depression Scale (anxiety); HADS-D, Hospital Anxiety and Depression Scale (depression); IEQ, Injustice Experience Questionnaire; EQ-5D, Euro Qol five-dimensional questionnaire. These data were analyzed using Spearman’s rank correlation coefficient test. The number of days that passed until insurance claim closure and the number of treatment visits were significantly associated with the IEQ score.

*Significance level was set at p < 0.05.

Results of the multiple regression analysis are shown in Tables 3 and 4. Three independent variables with p < 0.05 were analyzed in the multiple regression analysis for the number of days that passed until insurance claim closure: participants’ HADS-A, HADS-D, and IEQ scores. The number of days that passed until insurance claim closure was significantly associated with the IEQ scores (standardized beta value, 0.598; p < 0.001; Table 3).

**Table 3:** Multiple regression analysis for the number of days that passed until insurance claim closure.

Independent variables	B	SE	Beta	P value	R^2^
					0.358
IEQ	3.950	0.581	0.598	<0.001*	
HADS-A				0.076	
HADS-D				0.433	

B, nonstandard regression coefficient; SE, standard error, Beta, standardized regression coefficient; R^2^, multiple correlation coefficient adjusted for degrees of freedom, IEQ, Injustice Experience Questionnaire; HADS-A, Hospital Anxiety and Depression Scale (anxiety); HADS-D, Hospital Anxiety and Depression Scale (depression).

*Significance level was set at p < 0.05.

**Table 4:** Multiple regression analysis for the number of treatment visits.

Independent variables	B	SE	Beta	P value	R^2^
					0.537
IEQ	0.949	0.148	0.544	<0.001*	
HADS-D	1.494	0.425	0.299	0.001*	
Age				0.990	

B, nonstandard regression coefficient; SE, standard error, Beta, standardized regression coefficient; R^2^, multiple correlation coefficient adjusted for degrees of freedom, IEQ, Injustice Experience Questionnaire; HADS-D, Hospital Anxiety and Depression Scale (depression).

*Significance level was set at p < 0.05.

Further, three independent variables with p < 0.05 were analyzed in the multiple regression analysis for the number of treatment visits: participants’ age, HADS-D, and IEQ scores. The number of treatment visits was significantly associated with the IEQ (standardized beta value, 0.544; p < 0.001) and the HADS-D scores (standardized beta value, 0.299; p = 0.001; Table 4). Multi-collinearity was not observed for any of the independent variables tested in any of the regression analyses.

**Fig 3:**
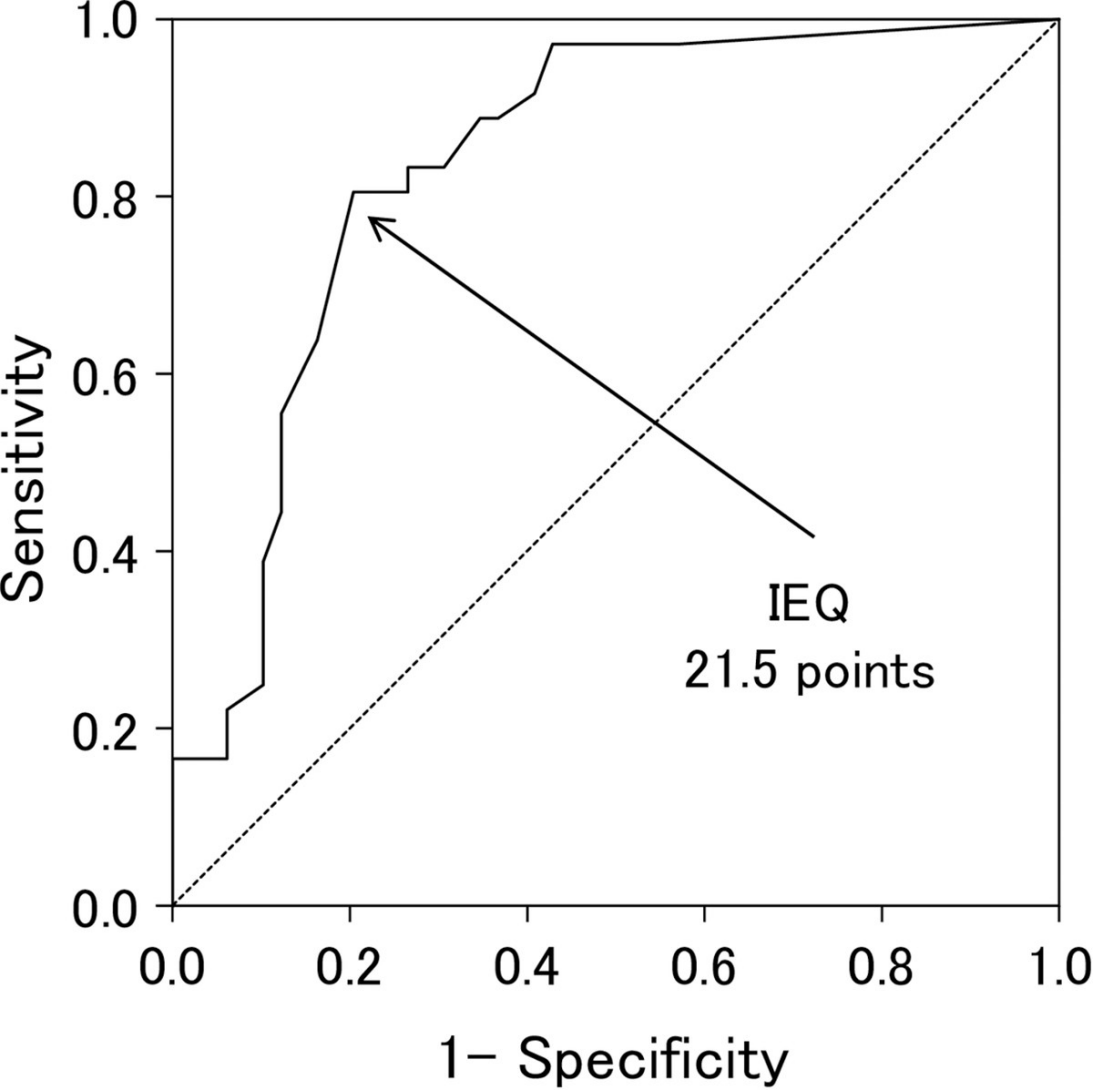
Receiver operating characteristic (ROC) curves of the IEQ scores for a prolonged number of days until insurance claim closure. The optimal cutoff point of the IEQ scores for a prolonged number of days until insurance claim closure was 21 and 22 points, respectively. IEQ, Injustice Experience Questionnaire. *Significance level was set at p < 0.05.

The value found in the area under the ROC curve of the IEQ scores for a prolonged number of days until insurance claim closure was 0.836 (95% Confidence Interval, 0.750 to 0.923; p < 0.001; Fig 3). The optimal cutoff point of the IEQ scores for a prolonged number of days until insurance claim closure was 21 and 22 points, respectively.

Comparisons between patients with a score on the IEQ of 21 or less (n = 46) and those with a score on the IEQ of 22 or higher (n = 39) are shown in Table 5. The latter, compared to the first, showed significantly higher scores for the NDI, HADS-A, and HADS-D scales.

**Table 5:** Comparison of data between patients with a score on the IEQ of 21 or less and those with a score on the IEQ of 22 or high.

	IEQ ≤ 21 (n = 46)	IEQ > 21 (n = 39)	p value
Women, n (%)	33 (72%)	23 (59%)	0.216
Age (years)	42 (17)	50 (20)	0.081
Injured body parts in addition to the neck			
Injured their heads, n (%)	12 (26%)	16 (41%)	0.144
Injured their torso, n (%)	18 (39%)	23 (59%)	0.068
Injured their limbs, n (%)	14 (30%)	15 (38%)	0.437
Large level of collision impact, n (%)	30 (65%)	19 (49%)	0.125
Presence of liability for an accident, n (%)	10 (22%)	7 (18%)	0.663
NRS (points)	5 [3–6]	5 [3–6]	0.129
NDI (points)	16 [10–22]	20 [13–24]	0.041*
HADS-A (points)	4 [2–7]	8 [5–11]	0.001*
HADS-D (points)	4 [2–5]	8 [4–12]	0.002*
IEQ (points)	2 [0–6]	29 [26–37]	<0.001*
EQ-5D (points)	0.71 [0.59–0.77]	0.66 [0.53–0.72]	0.152

NRS, Numeric Rating Scale; NDI, Neck Disability Index; HADS-A, Hospital Anxiety and Depression Scale (anxiety); HADS-D, Hospital Anxiety and Depression Scale (depression); IEQ, Injustice Experience Questionnaire; EQ-5D, Euro Qol five-dimensional questionnaire. Continuous data were expressed as means and standard deviations (SD). Non-continuous data (score data) were expressed as medians and interquartile ranges (IQR). Data from categorical variables are shown as number (%). The data were analyzed using Chi-square test and Mann-Whitney U test.

*Significance level was set at p < 0.05.

## Discussion

This study had two notable findings in patients with acute WAD: First, the IEQ scores among Japanese patients with acute WAD were higher than those among Canadian patients with the same condition; second, the IEQ scores during the early period after injury were associated with treatment terms among Japanese patients with WAD.

Evidence on the successful management of acute WAD remains at the low/very low level, [1] and the prognosis and incidence rates of this condition vary across different studies and countries. [3] For example, a study on WAD insurance claims in 1987 in the Canadian province of Quebec reported that 50% of the WAD claims were closed within 1 month. [30] Contrastingly, in Japan, more than half of the patients with acute WAD need treatment for more than 3 months. [5,31] The predictors of treatment terms are investigated among Japanese patients with WAD. [32,33] Reportedly, patients’ expectations toward the chronicity of their symptoms predicts a more passive coping style and a prevalence of the chronicity after WAD, and patients’ symptom expectations are different among countries. [18–21] Similarly, the present study showed a large number of days passed until insurance claim closure (mean, 106 days) and a high IEQ score (median, 16 points) among patients after acute WAD injury in Japan.

The treatment term is associated with post-injury symptoms and some psychosocial factors, not injury-related physical or mechanical factors in patients with acute WAD. [7] Our results suggested that treatment terms tended to associate with NDI psychosocial factors, but they did not relate to mechanical/physical factors (i.e. large collision impact)

When the injury has occurred as a result of another’s error or negligence, the victim might experience post-injury life with a sense of injustice. [34] This perceived injustice contributes to worsened pain-related variables (including working alliance, depression, and post-traumatic stress symptoms), which primarily owes to a consequential increase in feelings of isolation, disconnection with others, and anger-related emotions. [35–37] Perceived injustice also associates with reports of pain experiences and perceived difficulty after physical task performance. [38] Moreover, it induces poor progress in rehabilitation programs for musculoskeletal pain following injury. [37] Our study showed that the IEQ scores during the early period after injury was associated with treatment terms in Japanese patients with acute WAD. In another study, these same variables showed independent associations without multi-collinearity, although the IEQ scores associated with pain intensity, impairment, anxiety, and depression. [39] Further, treatment during the early period after injury is associated with the rate of recovery from WAD, [40], and an active behavioral physiotherapy intervention, including not only physiotherapy components but also behavioral components, has been developed. [41]

Populations with the short allele genotype for the serotonin-transporter-linked polymorphic region have greater anxiety-related personality traits, [42] depressive symptoms, [43] and neuroticism, [44] than those with the long allele genotype. Among Asians, the ratio for populations that are carriers of the short allele genotype revolve around 70–80%, whereas this same ratio for the European, North American, and the Australian populations revolve around 40–45%. [45] The present study also showed that the IEQ mean scores among the Japanese population were higher than those for the Canadian population.

Reportedly, the most appropriate cutoff score for the IEQ would be a score of 19 or higher, when related to the long-term disability of Canadian patients with subacute/chronic WAD; [12] since this study was the first to attempt to identify a cutoff score for the IEQ, the researchers deemed future research is necessary on the topic. [12] Following this recommendation, we showed that the optimal cutoff point for a prolonged number of days until insurance claim closure was an IEQ score of 22 or higher among Japanese patients with acute WAD. However, we still deem that further research is necessary to determine linguistic, cultural, or methodological influences that may have impacted these different cutoff points, which may also be related to the application of different language versions of pain-related questionnaires. [46]

Nevertheless, our study had several limitations. First, collected data on motor vehicle accidents, pain, and psychological variables were self-reported; that is, the impact of motor vehicle accidents was not assessed based on the actual force of the happening. Second, all data were collected by researchers who were not blinded. Third, the dose of analgesic medication administration was not recorded in detail. Fourth, the association with secondary gains owing to illness was not investigated. Finally, our sample included patients exclusively from a single medical center.

## Conclusions

The IEQ scores were elevated during the early period in patients with WAD in Japan. The IEQ scores during the early period after injury were associated with the number of days passed until insurance claim closure and treatment visits in patients with WAD in Japan.

## Supporting information

S1 Data (XLSX)
